# Supplementary material for: Multiscale stress dynamics in sheared liquid foams revealed by tomo-rheoscopy
Source: Nat Commun. 2025 Oct 16;16:9210. doi: 10.1038/s41467-025-64412-z (PMC12533193; doi:10.1038/s41467-025-64412-z)
Supplement: Supplementary file 1 — Supplementary Information [file 41467_2025_64412_MOESM1_ESM.pdf]

Supplementary Information

1 Yield stress fluids

The liquid foams we formulated are yield stress fluids, with a flow curve following Herschel-Bulkley model $\sigma_{ss} = \sigma_y + K\dot{\gamma}^n$. We verified such a behaviour by performing independent rheological measurements (plate-plate geometry, with 1.5 mm gap and 25 mm radius), on a wide range of shear rates 10^{-3} s$^{-1} < \dot{\gamma} < 1$ s^{-1} over foams produced at various liquid fractions with the same formulation and the same micro-fluidic setup. Thus, we were able to generate similar liquid foams (with comparable liquid fraction and bubble size). We show in Supplementary Figure 1 that indeed our liquid foams have a shear thinning behaviour with an exponent n close to 0.25, in quantitative agreement with the literature[1]. Furthermore, we superimpose in this figure the steady-state measurements obtained by our tomo-rheoscopy analysis at low shear rates (and the extrapolated values of the yield stress σ_y), showing a quantitative agreement between our different and independent rheological measurements.

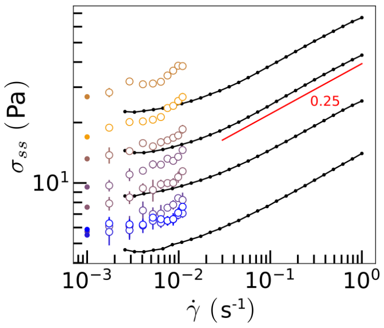

Supplementary Figure 1: Global steady-state stress measured (black line) on a wide range of shear rates 10^{-3} s$^{-1} < \dot{\gamma} < 1$ s^{-1} shows that our liquid foams of different liquid fractions follow Herschel-Bulkley model $\sigma_{ss} = \sigma_Y + K\dot{\gamma}^n$, with an exponent n close to 0.25 (red curve). Steady-state measurements obtained from our tomo-rheoscopy analysis at low shear rates, along with the extrapolated yield stress values σ_Y (colored circles), are superimposed.

2 Liquid fraction

We measure possible spatiotemporal variations of the liquid fraction for the eight experiments presented in the main text. Supplementary Figure 2a shows that the liquid fraction decreases slightly with time, probably owing to drainage and irradiation. Nonetheless, the relative decrease over the experimental runs remain weak, within at most 16% (Supplementary Table 1). Supplementary Figure 2b shows that the radial profile of the liquid fraction remains constant within 2%, consistent with the negligible centrifugal acceleration generated by the rotation. Supplementary Figure 2c shows that the liquid fraction decreases at increasing altitude, owing to gravitational drainage; however, here again, the relative variation over the gap remains within at most 14%.

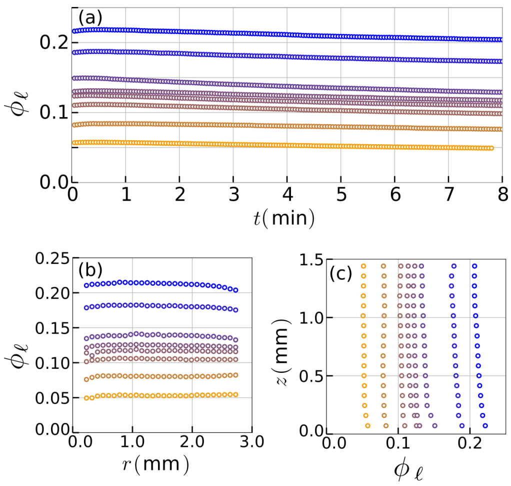

Supplementary Figure 2: (a) Time evolution of the liquid fraction averaged over the whole sample. (b) Time averaged liquid fraction as a function of the radial position r. (c) Time averaged liquid fraction as a function of the vertical position z .

Series	$\langle\phi_\ell\rangle$(%)	$\frac{\Delta\phi_\ell(t)}{\langle\phi_\ell\rangle}$(%)	$\frac{\delta\phi_\ell(r)}{\langle\phi_\ell\rangle}$(%)	$\frac{\Delta\phi_\ell(z)}{\langle\phi_\ell\rangle}$(%)
1	5.7	15.7	2.2	12.4
2	8.4	10.2	1.4	5.3
3	11.0	12.6	0.9	6.7
4	12.2	12.4	1.3	11.3
5	13.0	11.1	0.9	7.0
6	14.6	14.7	1.4	13.8
7	18.6	7.9	1.0	8.2
8	21.7	6.7	1.3	7.2

Supplementary Table 1: Liquid fraction inhomogeneities. Columns from left to right: number of the series; liquid fraction averaged over the whole sample and the whole duration of the runs; relative decrease of the space-averaged liquid fraction between the first and the last image (Supplementary Figure 2a); ratio of the standard deviation of the liquid fraction radial profile (Supplementary Figure 2b) to the average liquid fraction; relative increase of the liquid fraction between the highest and the lowest altitude in the foam sample (Supplementary Figure 2c).

3 Stress components

We present in Supplementary Figure 3, for the same reference experiment used for Supplementary Figure 2b of the main text (liquid fraction 8%, mean bubble radius 52 μm), all components of the deviatoric part of the stress as functions of the local deformation. Such a representation rescales measurements performed at different radii in the rheometer, as already shown in the mean text for $\sigma_{\theta z}$. The components $\sigma_{\theta r}$ and σ_{zr} show negligible deviations from zero, within 5% that of the dominant stress component $\sigma_{\theta z}$ (Supplementary Figures 3c and d). This is not the case for the normal stress components: σ_{rr} is slightly positive (Supplementary Figure 3a), but $\sigma_{\theta\theta}$ is significantly positive (Supplementary Figure 3b) and σ_{zz} significantly negative (Supplementary Figure 3c), their absolute values being around 34% that of the dominant stress component $\sigma_{\theta z}$. This means that our foams display a positive first normal stress difference $N_1 = \sigma_{\theta\theta} - \sigma_{zz}$, and a negative second normal stress difference $N_2 = \sigma_{zz} - \sigma_{rr}$. The fact that $N_1 > 0$ confirms previous rheometry measurements[2], but extends them at the local scale. To our knowledge, we provide the first experimental measurement of N_2, although its negative sign had been theoretically predicted[3].

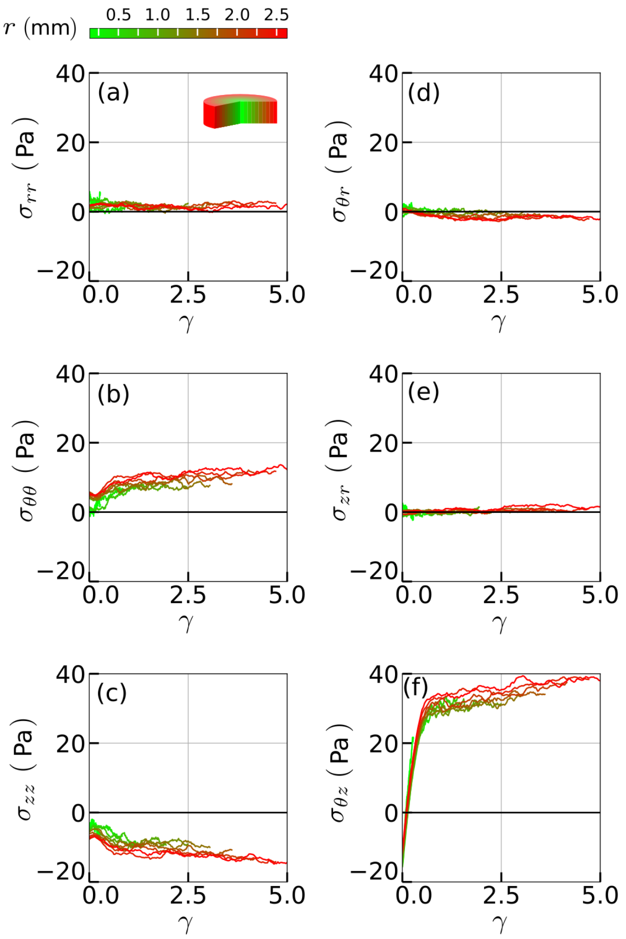

Supplementary Figure 3: Stress components as function of the local deformation γ. Panel f reproduces Figure 2b from the main text.

4 Statistical stress fluctuations

For a single bubble, the stress exhibits strong fluctuations as expected considering the disordered structure of the liquid foam. Those fluctuations can even be larger than the stress variation due to a topological rearrangement. However, thanks to our extensive data sets, we can average our stress measurements over a large number of bubbles or plastic events, significantly reducing the standard error and thus the uncertainty around the average stress values. This important aspect of our measurements is illustrated in Supplementary Figure 4, where the stress level measured at the scale of a single bubble is averaged over a larger number of bubbles $N_b = 3$ up to $N_b = 10000$.

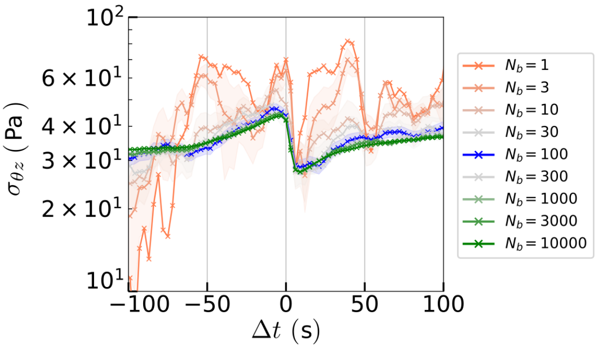

Supplementary Figure 4: Temporal variations of the stress measured around a T1 event occurring at $\Delta t = 0$, at the scale of a single bubble, and averaged over a larger number $N_b = 3$ up to $N_b = 10000$.

Such observation highlights the challenge — if not the impossibility — of detecting individual plastic events by monitoring stress on the scale of a single bubble. However, a reliable estimate of the stress with reduced uncertainty becomes apparent once the stress is averaged over approximately 100 bubbles

5 Typical T1 event

In the main manuscript, we present a typical example of a detected T1 event observed over three successive time steps. In Supplementary Figure 5, we further illustrate the position and orientation of this T1 event within the rheometer gap, over 60 time steps centered around the event. Three orthogonal projection views are shown in panels (a), (b), and (c). Corresponding Movies are also provided as Supplementary Information.

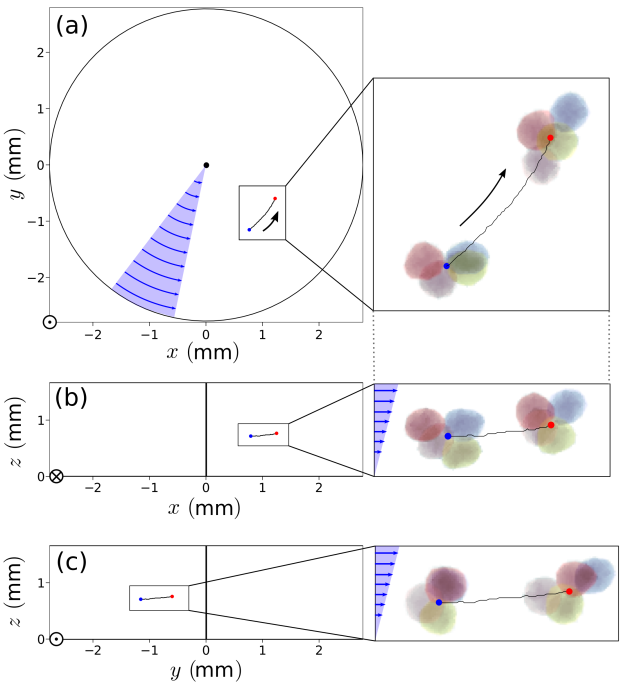

Supplementary Figure 5: Initial and final positions and orientations of the T1 event shown in the movies. The initial and final positions of the four-bubble cluster are indicated by blue and red dots, respectively. The trajectory is represented by a black line. The same color scheme is used to distinguish the four individual bubbles.

6 Orientation of the lost and new contacts

We present the orientations of all lost and new contacts for each of the eight experiments analysed. We measure the orientations from the natural directions of the cylindrical basis (Supplementary Figure 6a): α is the angle of the projection of the contact in the (θ, z) plane with respect to the azimuthal direction θ, and β is the angle of the projection of the contact in the (r, θ) plane, also with respect to θ. Since the orientation of a contact is irrelevant, we restrict α and β in the range between $-90°$ and $90°$. Supplementary Figure 6b shows that the distributions of α for lost and new contacts are unimodal, with a peak sharper at decreasing liquid fraction, and slightly sharper for the lost contacts as compared to the new contacts. The qualitative features of the two distributions are the same within a shift of around $90°$, consistently with the fact that for T1 events, lost and new contacts are in general orthogonal. The distribution of β is much broader (Supplementary Figure 6c), especially for the new contacts, with a small peak at $0°$ for the lost contacts and the lowest liquid fractions.

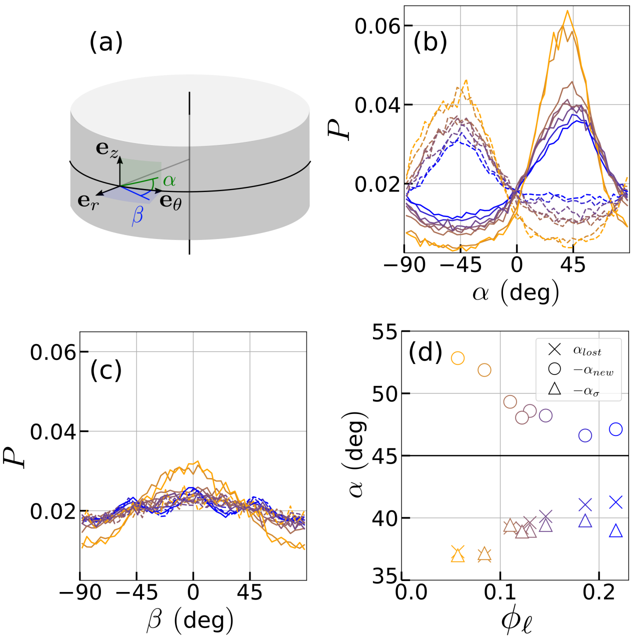

Supplementary Figure 6: (a) Sketch showing the definition of the orientation angles α and β. (b,c) Distribution of the orientations (b) α and (c) β for the lost contacts (plain curves), and the new contacts (dashed curves). (d) Plot of the peak of the distribution of α for the lost ($\times$) and new ($\circ$) contacts, and of the direction of maximal stress α_σ in the (θ, z) plane ($\triangle$), as functions of the liquid fraction.

The most interesting feature of the distributions is the location of the peak in α. For the lost contacts, the peak α_{lost} is close to $45°$, but consistently smaller, and slightly increases with the liquid fraction. It corresponds actually very closely (except for the largest liquid fractions) to the direction α_σ of the maximal elongational stress in the (θ, z) plane. While $\alpha_\sigma = 45°$ would be expected for pure

shear in the absence of first normal stress difference, the fact that it is lower than 45° is a clear signature of this first normal stress difference evidenced previously. The excellent agreement between α_{lost} and α_σ confirms that, in average, contact rearrangements are efficient means to release stress within the foam.

7 Fitted measures compared to Eshelby model

As mentioned in the main text, we compare the difference of the stress components between the two successive images between which a T1 event occurs to the stress field created by a quadrupolar displacement imposed on an elastic medium at the surface of a sphere of radius a. The imposed displacement writes:

$$\mathbf{u}^s = \boldsymbol{\varepsilon}^s \cdot \mathbf{x} \qquad \text{at } r = a, \tag{1}$$

with $\boldsymbol{\varepsilon}^s = \varepsilon^s(\mathbf{a} \otimes \mathbf{a} - \mathbf{b} \otimes \mathbf{b})$. This represents an imposed extension in the direction $\mathbf{a}$ and an equal imposed compression in the direction $\mathbf{b}$.

The equilibrium equation for an elastic medium of Young's modulus E and Poisson's ratio ν reduce to $\partial\sigma_{ij}/\partial x_j = 0$, where the indices represents components in the Cartesian basis $(\mathbf{a}, \mathbf{b}, \mathbf{c})$ and where Einstein's summation convention on repeated indices is used. Hooke's law gives the following relations between the stress and strain components u_{ij}:

$$\sigma_{ij} = \frac{E}{1+\nu}\left(u_{ij} + \frac{\nu}{1-2\nu} u_{kk}\delta_{ij}\right), \tag{2}$$

where the strain is related to the displacement by the equation $u_{ij} = (\partial u_i/\partial x_j + \partial u_j/\partial x_i)/2$. Hence, the equation of equilibrium written on the displacement components is:

$$\frac{\partial^2 u_i}{\partial x_j^2} + \frac{1}{1-2\nu}\frac{\partial^2 u_j}{\partial x_i\,\partial x_j} = 0.$$

The solution of this equation subjected to the boundary condition (1) can be obtained by several means, either following the procedure from the classical article by Eshelby[4], or by looking for a superposition of singularities[5]. The solution writes:

$$\begin{aligned} u_i &= \frac{a^3}{4-5\nu}\left\{\frac{1}{2}\left[5(1-2\nu) + 3\frac{a^2}{r^2}\right]\varepsilon^s_{ij}\frac{x_j}{r^3}\right. \\ &\quad \left. + \frac{15}{4}\left(1 - \frac{a^2}{r^2}\right)\varepsilon^s_{jk}\frac{x_i x_j x_k}{r^5}\right\}. \end{aligned}$$

Computing the strain components and inserting in (2) yields, after a straightforward but lengthy calculation:

$$\sigma_{ij} = \frac{Ea^3\varepsilon^s}{2(1+\nu)(4-5\nu)} g_{ij}, \tag{3}$$

with:

$$\begin{aligned} g_{xx} &= \left\{30\left(\nu - \frac{a^2}{r^2}\right)\frac{x^2}{r^5} + \left[5(1-2\nu) + 3\frac{a^2}{r^2}\right]\frac{1}{r^3}\right. \\ &\quad + \frac{15}{2}\left(-5 + 7\frac{a^2}{r^2}\right)\frac{(x^2-y^2)x^2}{r^7} \\ &\quad \left. + \frac{15}{2}\left(1 - \nu - \frac{a^2}{r^2}\right)\frac{x^2-y^2}{r^5}\right\}, \\ g_{yy} &= \left\{-30\left(\nu - \frac{a^2}{r^2}\right)\frac{y^2}{r^5} - \left[5(1-2\nu) + 3\frac{a^2}{r^2}\right]\frac{1}{r^3}\right. \\ &\quad + \frac{15}{2}\left(-5 + 7\frac{a^2}{r^2}\right)\frac{(x^2-y^2)y^2}{r^7} \\ &\quad \left. + \frac{15}{2}\left(1 - \nu - \frac{a^2}{r^2}\right)\frac{x^2-y^2}{r^5}\right\}, \\ g_{zz} &= \left[\frac{15}{2}\left(-5 + 7\frac{a^2}{r^2}\right)\frac{(x^2-y^2)z^2}{r^7}\right. \\ &\quad \left. + \frac{15}{2}\left(1 - \nu - \frac{a^2}{r^2}\right)\frac{x^2-y^2}{r^5}\right], \\ g_{xy} &= \frac{15}{2}\left(-5 + 7\frac{a^2}{r^2}\right)\frac{(x^2-y^2)xy}{r^7}, \\ g_{xz} &= \left[15\left(\nu - \frac{a^2}{r^2}\right)\frac{xz}{r^5}\right. \\ &\quad \left. + \frac{15}{2}\left(-5 + 7\frac{a^2}{r^2}\right)\frac{(x^2-y^2)xz}{r^7}\right], \\ g_{yz} &= \left[-15\left(\nu - \frac{a^2}{r^2}\right)\frac{yz}{r^5}\right. \\ &\quad \left. + \frac{15}{2}\left(-5 + 7\frac{a^2}{r^2}\right)\frac{(x^2-y^2)yz}{r^7}\right], \end{aligned} \tag{4}$$

where indices x, y and z indicate here components along the respective directions $\mathbf{a}$, $\mathbf{b}$ and $\mathbf{c}$).

To compare these predictions with our measurements, we focus on the reference experiment ($\phi_\ell = 8.4\%$, $R_{32} = 57.8$ μm). We impose $\nu = 1/2$ assuming foam is locally incompressible (because its shear modulus is much lower than its bulk modulus, of order the ambient pressure 10^5 Pa), $E = 2G(1+\nu) = 351$ Pa from the rescaled local shear stress $\sigma_{\theta z}$ elastic slope, and $a = 2R_{32} = 115.6\mu$m, and treat ε^s as a single fitting parameter. This procedure gives, for instance, the theoretical stress maps of Figure 4 in the main text, which are in remarkable qualitative agreement with our measurements.

To provide a more quantitative comparison, we plot in Supplementary Figure 7 the angular dependence of all stress component variations in the planes ab, ac and bc obtained both from experiments and from the theoretical prediction, at various radial distances from the centre of the T1 event. This yields a total of eighteen plots, which confirm the excellent agreement between our experimental data and Eshelby's prediction. In practice, ε^s is inferred by determining the best proportionality relation when plotting all experimentally measured components $\Delta\sigma_{ij}$ as a function of $\frac{Ea^3}{2(1+\nu)(4-5\nu)}g_{ij}$, as predicted by Eq. (3). As shown in Supplementary Figure 8, this proportionality relation holds, and the proportionality coefficient provides the value of ε^s. For our reference experiment, we obtain the value, $\varepsilon^s = 0.068 \pm 0.002$.

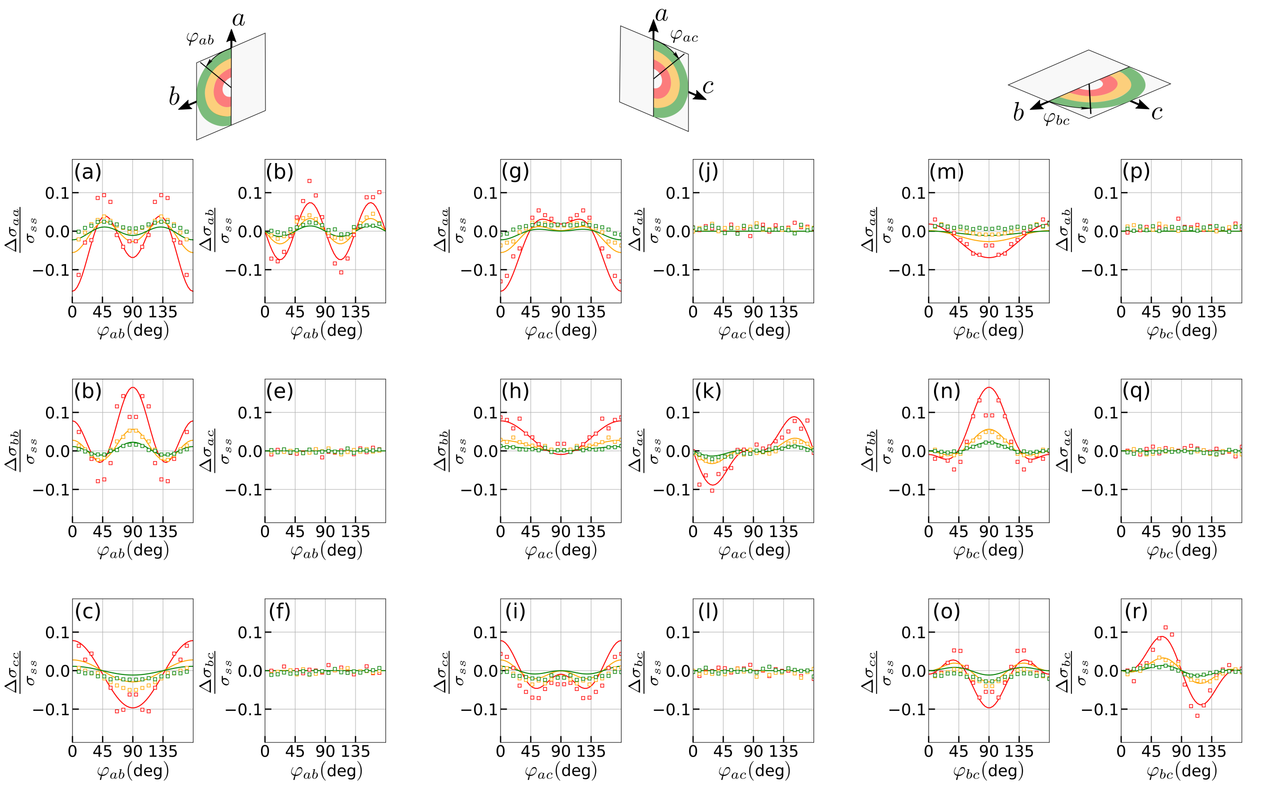

Supplementary Figure 7: Plots for the reference experiment of the variations of the six components of the stress, rescaled by the steady-state shear stress, as functions of the angles φ_{ab} in the (a, b) plane (panels a-f), φ_{ac} in the (a, c) plane (panels g-l) and φ_{bc} in the (b, c) plane (panels m-r). Symbols are experimental data and curves from Eqs. (4), with $\varepsilon^s = 0.068$ as a single fitting parameter. Colours indicate different distances from the T1 centre: in red, from $2R_{32}$ to $4R_{32}$; in orange, from $4R_{32}$ to $6R_{32}$; in green, from $6R_{32}$ to $8R_{32}$. The theoretical curves are estimated at $r = 3R_{32}$ (red), $5R_{32}$ (orange) and $7R_{32}$ (green).

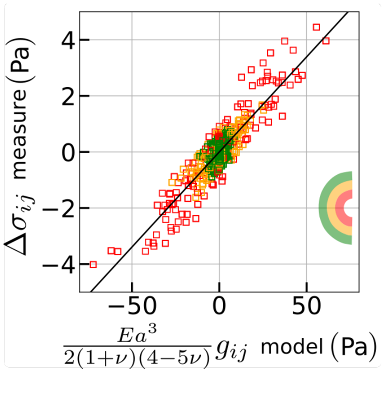

Supplementary Figure 8: Validation of Eshelby model (Eq. (3)) considering all $\Delta\sigma_{ij}$ components at various distances (using the same color scheme as in Supplementary Figure 7). The fitting parameter $\varepsilon^s = 0.068 \pm 0.002$ is inferred from the coefficient of the best fit based on a proportionality relation.

We have performed the same analysis for all experiments at different liquid fractions, yielding similar very good agreements. This fitting parameter ε^s decreases at increasing liquid fraction (Supplementary Figure 9a). On the other hand, ε^s appears systematically lower than the yield strain (Supplementary Figure 9b). Nevertheless, we observed that the duration of T1 events systematically decreases with increasing liquid fraction, ranging from approximately 12 seconds for dry foams to 6 seconds for wet foams. This significantly exceeds the 3-second interval used here to evaluate stress variations, by a factor of 2 to 4. Indeed, the cumulative deformation estimated over the measured duration of the T1 events yields a value remarkably close to the yield strain, γ_Y.

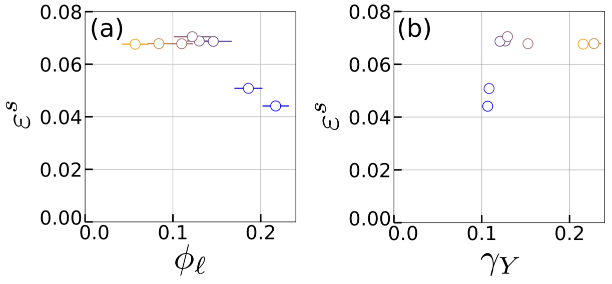

Supplementary Figure 9: The fitting parameter ε^s as a function of the liquid fraction ϕ_ℓ (a) and the yield strain γ_Y (b), respectively.

References

[1] N. D. Denkov, S. Tcholakova, K. Golemanov, K. P. Ananthpadmanabhan and A. Lips, Soft Matter, 2009, **5**, 3389–3408.

[2] V. Labiausse, R. Höhler and S. Cohen-Addad, J. Rheol., 2007, **51**, 479–492.

[3] R. Höhler, S. Cohen-Addad and V. Labiausse, J. Rheol., 2004, **48**, 679–690.

[4] J. D. Eshelby, Proc. R. Soc. A, 1957, **214**, 376–396.

[5] L. D. Landau and E. M. Lifshitz, Theory of Elasticity, Butterworth–Heinemann, 3rd edn., 1986.
