# Supplementary material for: Multiscale stress dynamics in sheared liquid foams revealed by tomo-rheoscopy
Source: Nat Commun. 2025 Oct 16;16:9210. doi: 10.1038/s41467-025-64412-z (PMC12533193; doi:10.1038/s41467-025-64412-z)
Supplement: Supplementary file 2 — Description of Additional Supplementary Files [file 41467_2025_64412_MOESM2_ESM.pdf]

Description of Additional Supplementary Files

File Name: Supplementary Movie 1
Description: The movie shows an experiment conducted with series 1, observed in the $\{\mathbf{e}_\theta,\ \mathbf{e}_z\}$ plane at $r = R/2 = 1.375$ mm. The scale is indicated by the height of the foam sample, which is 1.5 mm.

File Name: Supplementary Movie 2
Description: The movie shows the same conditions but with series 6, which has a higher liquid fraction than series 1.

File Name: Supplementary Movie 3
Description: The movie shows an experiment conducted with series 1, observed in the $\{\mathbf{e}_r,\ \mathbf{e}_\theta\}$ plane at $z = h/2 = 0.75$ mm. The scale is indicated by the radius of the foam sample, which is 2.75 mm.

File Name: Supplementary Movie 4
Description: The movie shows the same conditions but with series 6, which has a higher liquid fraction than series 1.

All cross-sectional movies have been accelerated by a factor of 60 relative to real time.

File Name: Supplementary Movie 5
Description: Movie showing the rearrangement dynamics of a detected T1 event, captured from front view. The video is played at 10 times the real-time speed.

File Name: Supplementary Movie 6
Description: Movie showing the rearrangement dynamics of the same detected T1 event, captured from back view. The video is played at 10 times the real-time speed.
